# Single or Blended Application of Non-Microbial Plant-Based Biostimulants and *Trichoderma atroviride* as a New Strategy to Enhance Greenhouse Cherry Tomato Performance

**DOI:** 10.3390/plants13213048

**Published:** 2024-10-31

**Authors:** Lorena Vultaggio, Michele Ciriello, Emanuela Campana, Pietro Bellitto, Beppe Benedetto Consentino, Youssef Rouphael, Giuseppe Colla, Fabiana Mancuso, Salvatore La Bella, Simona Napoli, Leo Sabatino

**Affiliations:** 1 Dipartimento Scienze Agrarie, Alimentari e Forestali, Università di Palermo, Viale delle Scienze, 90128 Palermo, Italy; lorena.vultaggio@unipa.it (L.V.); pietro.bellitto@unipa.it (P.B.); fabiana.mancuso@you.unipa.it (F.M.); salvatore.labella@unipa.it (S.L.B.); leo.sabatino@unipa.it (L.S.); 2 Department of Agricultural Sciences, University of Naples Federico II, 80055 Portici, Italy; michele.ciriello@unina.it (M.C.); emanuela.campana@unina.it (E.C.); youssef.rouphael@unina.it (Y.R.); 3 Department of Agriculture and Forest Sciences, University of Tuscia, 01100 Viterbo, Italy; giucolla@uinitus.it; 4 Research Consortium for the Development of Innovative Agro-Environmental Systems (Corissia), Via della Libertà 203, 90143 Palermo, Italy

**Keywords:** biostimulant synergy, greenhouse cultivation, microbial biostimulants, non-microbial biostimulants, *Solanum lycopersicum* L.

## Abstract

The need to increase yield and enhance the sustainability of crop production systems has led to the development and employment of natural products, such as plant biostimulants. In recent years, a number of reports have researched the effects of biostimulants on plant performance; however, few studies have focused on the mutual application of microbial and/or non-microbial biostimulants. This research, conducted in the framework of the SO.MI.PR.O.N regional project, aimed to investigate the single or mutual application of three biostimulants, a tropical plant extract (PE), a vegetal protein hydrolysate (PH), and *Trichoderma atroviride*, on ‘Creativo’ F1 cherry tomato plants cultivated during two growing cycles (2022–2023 and 2023–2024). Our results showed that plants treated with the combination Tricho + PE + PH had statistically significant higher fresh shoot biomass (+64.2%, 1647.0 g plant^−1^), total fruit production (+37.9%, 1902.5 g plant^−1^), marketable fruit production (+52.9%, 1778.5 g plant^−1^), and average weight of marketable fruits (+53.1%, 17.0 g) compared to control plants (untreated plants). Furthermore, biostimulant treatments, especially *T. atroviride*, variably enhanced cherry tomato fruits’ qualitative traits, such as firmness, total soluble solids, ascorbic acid, lycopene, and total polyphenols compared to control plants. Overall, the best combinations to increase tomato fruit qualitative features were PE + PH, Tricho + PE, and Tricho + PH. From an economic point of view, the best treatment for achieving the highest net return was PE. This study underlines that biostimulant features (yield, qualitative aspects, and economic profitability) can be supported through the application of specific biostimulant combinations.

## 1. Introduction

Tomato (*Solanum lycopersicum* L.) is one of the most consumed vegetables at the global level and its fruits are consumed fresh or processed (soups, juices, sauces, etc.) [1]. Tomato is a notable source of bioactive components efficient of granting appropriate health benefits in humans, such as carotenoids (primarily lycopene and β-carotene), ascorbic acid, flavonoids, and phenols, displaying antioxidant and antiradical activity [2,3,4,5]. In the Italian diet, tomato fruits can be considered as a primary source of lycopene, β-carotene, and polyphenols [6,7,8], and, accompanied by a restricted caloric supply, tomato fruits can be considered as a natural functional food. As reported by the FAO Statistical Database [9] (http://www.fao.org, accessed on 17 July 2024), the estimated total world production of tomato is approximately 190 million tonnes, with China contributing 31.6% to world production. In Europe, Italy is the main tomato producer (6,000,000 t in 2021), while the Apulia region is the leading producer in Italy (more than 2,000,000 t), followed by Sicily (632,038 t) [10].

Tomato is commonly grown in intensive monoculture systems, especially when it is cultivated under a protected or controlled environment [11,12,13]. As greenhouse cultivation has a prominent impact on agro-ecosystems, efforts are currently focused on finding sustainable and pioneering agronomic tools that could provide good yield and crop quality and, simultaneously, decrease the horticultural sector’s impact on the environment [14,15,16,17,18,19,20,21]. Concomitantly, consumers’ growing concern about ecological and well-being topics drives them to eat healthy and accessible vegetables cultivated via eco-friendly and sustainable cultivation practices [22,23,24,25]. Thus, contemporary horticulture is exposed to the strict reduction in synthetic pesticide and fertilizer use [26,27]. There are reports on an eclectic assortment of vegetables that, once exposed to the foliar or root supply of non-microbial biostimulants, such as vegetal-based protein hydrolysates (V-PHs) or plant extracts (PEs), manifest in enhanced plant metabolism, enhanced mineral uptake through the stimulation and accumulation of the phytochemical’s biosynthesis, and increased abiotic distress tolerance [28,29]. This in turn increases the yield of vegetables [30,31].

The upsurge in crop yield encouraged by non-microbial biostimulants (V-PHs and PEs) under favorable, sub- or supra-optimal, or unfavorable growing environments could be related to various direct and indirect collaborative physiological phenomena, comprising the elicitation of enzymatic actions, triggering of hormone-like activity, and modulation of the plant root system, which leads to advanced absorption capacity of water and nutrients [32,33]. Simultaneously, within the biostimulants panorama, microbial biostimulants, such as plant growth-promoting rhizobacteria (PGPR), arbuscular mycorrhizal fungi (AM), or *Trichoderma* spp., substantially promote plant growth and productivity, in addition to nutritional and functional quality [34,35,36,37,38]. *Trichoderma* spp. are versatile fungi with diverse effective tasks, making them a suggested tool to boost horticultural sustainability. These acknowledgments may include the capacity of *Trichoderma* spp. as biocontrol agents, plant growth elicitors, and biofertilizers [39,40,41,42,43,44]. Likewise, *Trichoderma* spp. might reduce plant abiotic stress by triggering endogenous mechanisms controlled by plant hormones and alterations in the host plant’s metabolism [45,46,47].

Notwithstanding relevant studies on the influences of microbial and non-microbial biostimulants on vegetables, an improved comprehension of the merging application of microbial and/or non-microbial biostimulants is essential to enhance horticultural sustainability and resilience [48,49,50,51]. In this regard—to the best of our knowledge—there is no scientific indication of the effect of the joint application of *T. atroviride* and two non-microbial biostimulants, namely a V-PH and a PE, on the plant growth, yield, and fruit quality features of cherry tomato. The present research wants to fill the research gap regarding the effects of microbial and non-microbial biostimulants’ mutual application (additive, synergistic, or antagonistic) on tomato plants. Thus, a study was conducted with the aim of explaining the influences of three biostimulants (*T. atroviride*, a V-PH, and a PE)—supplied alone or in a blend—on the performance and economic profitability of an F_1_ cherry tomato genotype. The results of this study will help to understand the possible interactions between different classes of biostimulants for the modulation of cherry tomato plant performance.

## 2. Results

### 2.1. Analysis of Variance (ANOVA)

Table 1 shows the Analysis of Variance (ANOVA) output for all recorded parameters.

For all parameters, ANOVA did not point out a statistically significant influence of year; indeed, the *p*-values ranged from 0.398 to 0.959. Contrariwise, biostimulant treatments had a highly significant effect (*p*-value ≤ 0.001) on tomato productive and qualitative traits. Something that is noteworthy, as shown in Table 1, is the significant influence of the interaction between year and biostimulant recorded for fresh shoot biomass (*p*-value ≤ 0.001), titratable acidity (*p*-value ≤ 0.001), ascorbic acid (*p*-value = 0.013), and lycopene (*p*-value = 0.004).

### 2.2. Greenhouse Temperatures, Fresh Shoot Biomass, and Fruit Production Traits

Overall, during the first cultivation year (2022–2023), lower maximum and minimum temperatures were recorded as compared to the second year; in particular, lower maximum temperatures were recorded at the beginning of the growing cycle (first month) and in the final phase of the cultivation period (last two months), whereas lower minimun temperatures were observed from mid-January to mid-February.

**Figure 1:**
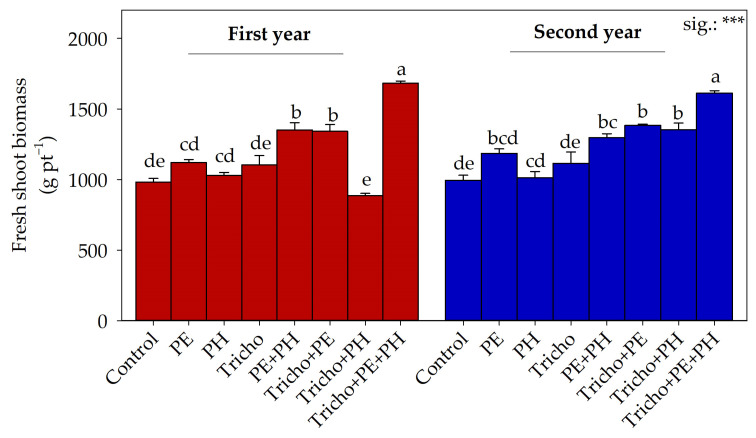
Fresh shoot biomass as influenced by cultivation year and biostimulant interaction. Separation of Y × B interaction was performed using Tukey’s HSD test (*p* ≤ 0.05). ***: significant at *p* ≤ 0.001. First year: 2022–2023; second year: 2023–2024; Control: non −treated plants; PE: tropical plant extract; PH: legume-derived protein hydrolysate; Tricho: *Trichoderma atroviride*. Different letters indicate significantly different means. Bars indicate the standard error.

The biostimulant × year interaction statistically influenced fresh shoot biomass (Figure 1).

Plants supplied with Tricho + PE + PH cultivated both during the first or second year had the highest values, followed by those recorded in the plants treated with Tricho + PE grown in the first or in the second year, those supplied with PE + PH grown during the first year, and those treated with Tricho + PH cultivated during the second year. The lowest fresh shoot biomass was found in tomato plants supplied with Tricho + PH grown in the first year. ANOVA showed that year (Y) did not statistically affect productive features (Table 2).

The application of PE, PH, Tricho, and all of their combinations positively affected total fruit production, marketable fruit production, and average weight of marketable fruit (Table 2). Cherry tomato plants treated with PE or with Tricho + PE + PH had the highest total fruit production, followed by those sprayed with PE + PH. The lowest total fruit production values were recorded in control plots (Table 2). The biostimulant treatments, accomplishing the highest increments in terms of marketable fruit production were PE, PE + PH, or the combinations of all biostimulants (Tricho + PE + PH). Control plots showed the lowest values. Plants from plots treated with PE had the highest number of marketable fruits. The PE + PH and Tricho + PE + PH combinations led to a significant higher average weight of marketable fruits compared to the control plants (Table 2). In comparison, control plants had the lowest average weight of marketable fruits.

### 2.3. Fruit Quality Features

For firmness, TSS, and fruit dry matter, ANOVA did not highlight a significant effect of year (Y) (Table 3).

Contrariwise, the results underline that biostimulant supply statistically affects the firmness, TSS, and dry matter of tomato fruits. Overall, *T. atroviride*-treated plots exhibited the highest firmness values (+33% compared to the control plots). The lowest firmness was obtained in tomatoes from control plants and in those from plants treated with all biostimulants (Table 3). For fruit TSS, our data showed that fruits from PH or Tricho plots showed an increase of 38.6% compared to tomato fruits from control plants, which in turn exhibited the lowest value. The highest fruit dry matter was recorded in tomato fruits from control and Tricho + PH-treated plots, whereas plants treated with the combinations of the three biostimulants (Tricho + PE + PH) showed the lowest values (Table 3).

**Figure 2:**
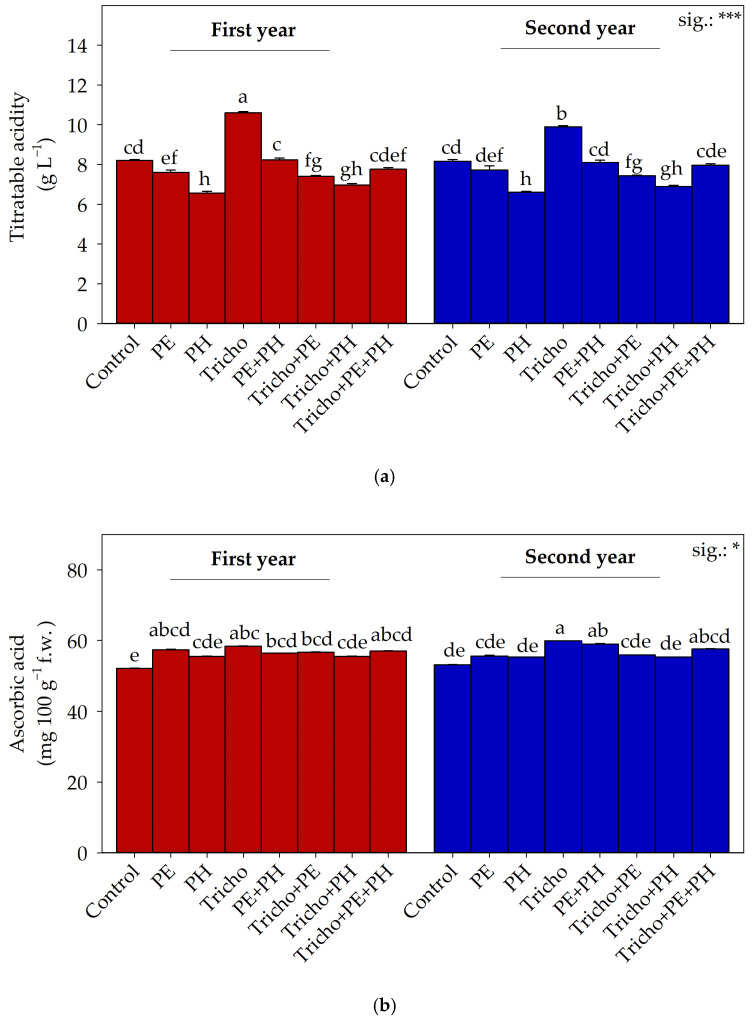
Titratable acidity (**a**) and ascorbic acid (**b**) as influenced by cultivation year and biostimulant interaction. Separation of Y × B interaction was performed using Tukey’s HSD test (*p* ≤ 0.05). First year: 2022–2023; second year: 2023–2024; Control: non-treated plants; PE: tropical plant extract; PH: legume-derived protein hydrolysate; Tricho: *Trichoderma atroviride*. Bars indicate standard error. Different letters indicate significantly different means. *: significant at *p* ≤ 0.05; ***: significant at *p* ≤ 0.001.

ANOVA underlined that the biostimulant × year interaction significantly influenced titratable acidity and ascorbic acid (Figure 2). Concerning titratable acidity, fruits from Tricho-inoculated plants cultivated in the first year had the highest values, followed by those treated with the same biostimulant and cultivated during the second year (Figure 2a). Plants supplied with the PH revealed the lowest values, independently of the cultivation year.

Fruits from Tricho-treated plants grown during the second year had the highest ascorbic acid concentration, followed by those harvested from plants treated with the PE + PH combination grown during the second year. Fruits from control plots cultivated in the first year had the lowest values (Figure 2b).

**Figure 3:**
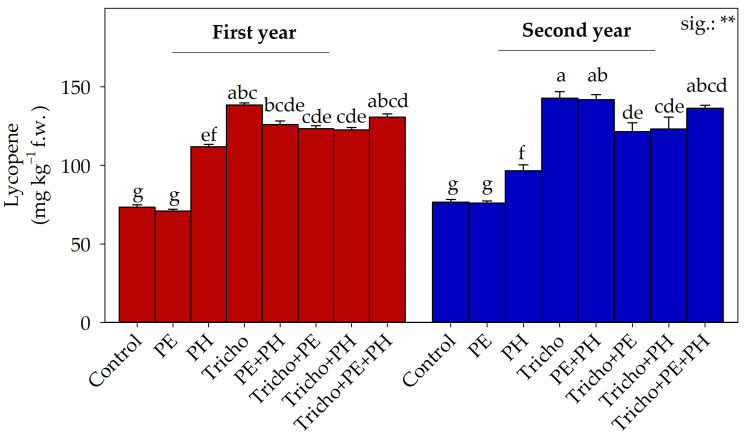
Lycopene as influenced by cultivation year and biostimulant interaction. Separation of Y × B interaction was performed using Tukey’s HSD test (*p* ≤ 0.05). First year: 2022–2023; second year: 2023–2024; Control: non-treated plants; PE: tropical plant extract; PH: legume-derived protein hydrolysate; Tricho: *Trichoderma atroviride*. Bars indicate the standard error. Different letters indicate significantly different means. **: significant at *p* ≤ 0.01.

For lycopene, ANOVA evidenced a significant influence of Y × B interaction. The highest values were found in fruits from plants grown in the second year and supplied with Tricho, whereas the lowest values were observed in control or PE-treated plots cultivated during the first or second year (Figure 3).

**Figure 4:**
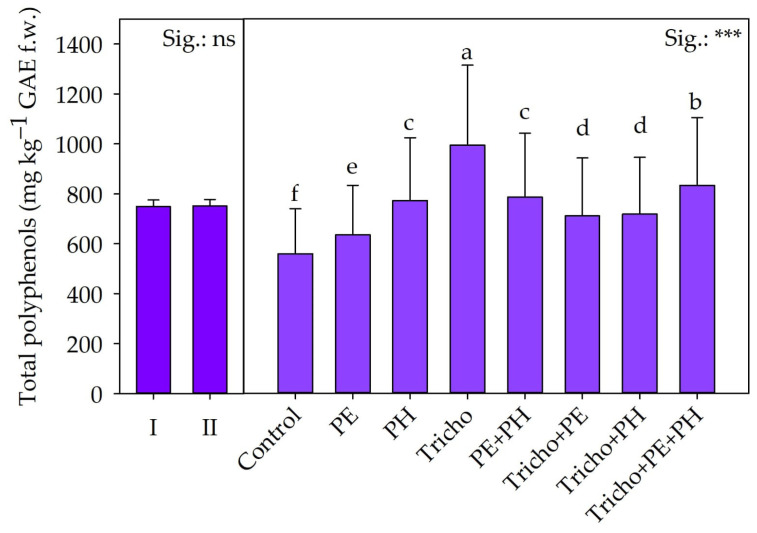
Total polyphenols as influenced by cultivation year and biostimulants. Separation of the mean biostimulant (B) effect was performed using Tukey’s HSD test (*p* ≤ 0.05) while the mean Y effect was compared using the *t*-test. ***: significant at *p* ≤ 0.001. First year: 2022–2023; second year: 2023–2024; Control: non-treated plants; PE: tropical plant extract; PH: legume-derived protein hydrolysate; Tricho: *Trichoderma atroviride*. Bars indicate the standard error. Different letters indicate significantly different means.

Statistical analysis revealed that year did not affect total polyphenol concentration (Figure 4).

Inversely, biostimulant supply statistically affected total polyphenols in tomato fruits (Table 3). Fruits from Tricho-treated plants showed the highest polyphenol content (+78%), followed by those treated with Tricho + PE + PH (+49%). The lowest polyphenol concentration was found in fruits from control plots.

### 2.4. Heat-Map Analysis

**Figure 5:**
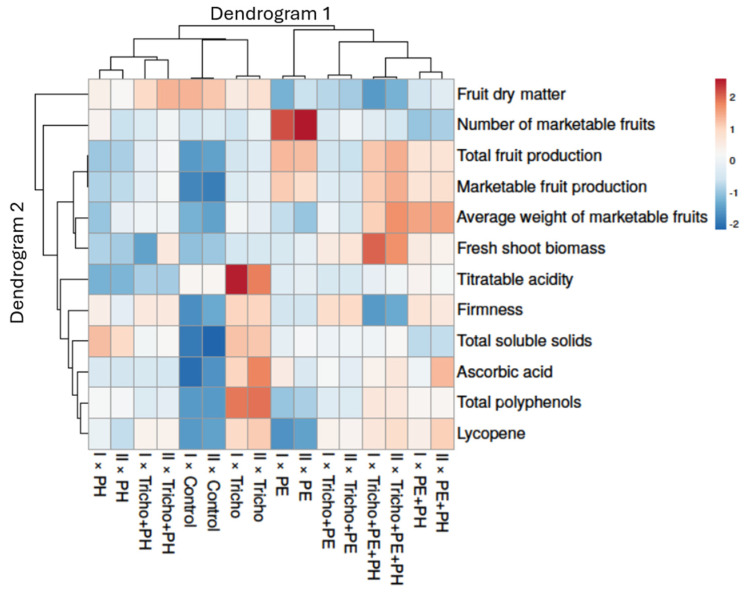
Heat-map analysis including all cherry tomato plant traits in response to cultivation year and biostimulant treatments. The heat-map figure was created using the https://biit.cs.ut.ee/clustvis/ online program (accessed on 1 August 2024) package. I: first cultivation year; II: second cultivation year; Control: non-treated plants; PE: tropical plant extract; PH: legume-derived protein hydrolysate; Tricho: *Trichoderma atroviride*.

To summarize the impact of all experimental factors on cherry tomato plants, a heat-map analysis, involving the entire data set, was performed (Figure 5).

The heat-map analysis showed two dendrograms (Dendrogram 1 and Dendrogram 2). The first, located on the top, contains the combinations between year (Y) and biostimulant (B). Dendrogram 2, located on the left side of the graphical analysis, includes the dependent variables, which affected the distribution.

For fruit dry matter, values similar to the control were detected in Tricho + PH and Tricho treatments. Regarding the number of marketable fruits, all treatments showed similar values to the control, with the exception of PE treatment. Contrariwise, for total fruit production, marketable fruit production, and the average weight of marketable fruits, all treatments revealed dissimilar values compared with the control, excluding PH treatment. For fresh shoot biomass, PH, Tricho, and PE treatments had similar values compared to the control. Moreover, for titratable acidity and firmness, the heat-map analysis revealed that Tricho + PE + PH showed analogous values to the control. However, for firmness, PE + PH treatment also had values comparable to the control. The treatment PE + PH—for total soluble solids—gave similar values to the control, whereas, for ascorbic acid, the heat-map detected that all treatments were different from the control. Lastly, for total polyphenols and lycopene, data similar to the control were detected in PE treatment.

### 2.5. Partial Budget Analysis

The biostimulant effects on the partial budget analysis of “Creativo F” cherry tomato are presented in Table 4.

All biostimulants, applied alone or in combination, increased the profitability of the cherry tomato crop. The economic assessment showed that the highest added gross return was observed in plants treated with PE (28,154.4 EUR ha^−1^), followed by those treated with PE + PH (23,963.6 EUR ha^−1^), which in turn was higher than those exposed to PE + PH + Tricho (20,009.5 EUR ha^−1^). Overall, our results showed that the greatest net economic benefit was related to yield increase. In particular, the use of PE was the most profitable treatment.

## 3. Discussion

Overall, data on most tomato productive and growth traits revealed a positive effect of the three biostimulants. This is in accordance with the results of Colla et al. [32], who studied the effect of different biostimulants (plant extract and protein hydrolysate) on tomato plants and reported an increase in productive features. Moreover, our data corroborate those of Sani et al. [51], who stated that tomato plants treated with *Trichoderma* spp. and grown under organic conditions significantly enhanced growth and yield. Colla et al. [52] reported that the mechanism behind the stimulatory effect of PE might be related to its phytohormone and signaling compound content, which increases the plant photosynthesis rate and, consequently, productive features. Furthermore, the same authors reported that the beneficial effect of PE on plant growth and yield could be linked to its ability to increase plant nutrient uptake via the modulation of plant root expansion. The positive effect on tomato fresh shoot biomass and productive parameters recorded in plants treated with the PH can also be associated with well-known hormone-like activity, which in turn stimulates the expression of some genes involved in cell division and expansion [53]. In comparison, De Palma et al. [54] reported that *Trichoderma* genera are implicated in the upregulation of many proteins involved in photosynthesis, with repercussions for photosynthetic rate and, accordingly, plant yield. *Trichoderma* spp. are also implicated in numerous biological and chemical activities in the rhizosphere, such as the increase in nutrient availability and the increase in root growth [55]. In addition, various authors [56,57] state that *Trichoderma* spp. can produce secondary metabolites with hormone-like activity, small peptides, and volatile organic compounds, which are known for their biostimulatory effects. Remarkably, our results showed a positive effect of biostimulants on productive traits, especially when they were applied in combination. For total fruit production, marketable fruit production, and the average weight of marketable fruits, all biostimulants (Tricho + PE + PH) had an additive effect. However, the number of marketable fruits did not show a similar trend. Moreover, the positive additive effect between PE and PH was also documented for marketable fruit production and the average weight of marketable fruits. Even for fresh shoot biomass, the combination of all biostimulants showed synergistic action. However, in this case, the year of cultivation also had a significant effect on plant response to biostimulant application.

Fruit firmness was enhanced by biostimulant application. Likewise, Vultaggio et al. [34], investigating the effect of biostimulants on woodland strawberry, revealed an increase in firmness in fruits from plants inoculated with *Trichoderma*. Contrariwise, Soteriou et al. [56] reported that a vegetal PH did not have a significant effect on watermelon fruit firmness. Since contrasting results are reported in the literature, we can speculate that the variation in this parameter to PH application depends on the plant species. However, the positive effect recorded on fruit firmness in plants treated with *T. atroviride* can be associated with its capacity to make calcium more available for plants [57]. We also recorded a synergistic effect of biostimulants on fruit firmness, with no statistical differences among PE + PH, Tricho + PE, and Tricho + PH. However, when all biostimulants were combined (Tricho + PE + PH), no significant variation from the control was recorded. This behavior can be linked to an excessive effect of biostimulants in increasing plant water uptake, which, in turn, can cause a reduction in fruit firmness. Thus, in this case, the mutual application of all biostimulants had a counterproductive effect.

Our total soluble solids (TSS) data concur with those of Sani et al. [51], who found an increase in fruit TSS when plants were inoculated with *Trichoderma* spp. The effect exerted by PE and PH on fruit TSS can be linked to their amino acid and peptide content, which have a positive effect on plant primary metabolism [58]. Furthermore, it seems that PE and PH biostimulants promote photosynthesis rates, with a positive impact on soluble solids biosynthesis [52]. The *T. atroviride* effect on TSS could be the consequence of increased plant mineral uptake, which in turn elicited the production of soluble solids [58]. However, no synergistic effect among the biostimulants was recorded for TSS.

Fruit dry matter was reduced by biostimulant application, especially when all of them were combined. Since it has been reported that PE, PH, and *T. atroviride* application can increase plant water uptake [52,54], we can assume that the decrease in fruit dry matter could be the consequence of higher water content in fruit tissues compared to the control. These data may also be partially related to firmness since we noticed that fruit dry matter decreases when the biostimulant applications were combined.

Our data on ascorbic acid corroborate those of Sani et al. [51], who reported an upsurge of ascorbic acid in fruits from plants inoculated with *T. atroviride*. The higher values of ascorbic acid recorded in fruits from plants cultivated in the second-year experiment can be related to the higher temperature recorded. Indeed, since ascorbic acid production increases for ROS detoxification when plants are under stress [59] and considering that the temperatures of the second year were higher than those of the first cultivation cycle, we can hypothesize that this response is a direct temperature-related effect. Regarding the biostimulants’ effect on ascorbic acid, there are reports on the influence of PE, PH, and *Trichoderma* spp. applications on plant secondary metabolism elicitation [55].

Titratable acidity was reduced by PE and PH application and enhanced by *T. atroviride* inoculation. Our data are not in line with those of Abdelkader et al. [60], who reported no significant effect of biostimulants on tomato acidity. Since the genotype used in this study is different from that employed by Abdelkader et al. [60] and considering that the growing conditions were different, we can hypothesize that the effects of these biostimulants on tomato acidity are both environmental and plant genetic dependent. However, our data on *T. atroviride*’s impact on titratable acidity corroborate those of Ruiz-Cisneros et al. [61], who noted an improvement in fruits from inoculated plants. The outcome may be related to the fruit size increase recorded in inoculated plants since, as reported by Tigist et al. [62], acidity increases with the mass of the fruits.

Lycopene in tomato fruits was increased by PH and *T. atroviride* application. Our data agree with those of Choi et al. [63], who, studying the impact of a PH on greenhouse lettuce and tomato performance, reported an increase in fruit lycopene concentration when plants were supplied with the biostimulant. This could be the result of the activation of physiological and molecular mechanisms [64]. Furthermore, our data agree with those of Sani et al. [51], who reported an increase in fruit lycopene concentration when plants were inoculated with *T. atroviride* compared to control plants. As suggested by López-Bucio et al. [58], *T. atroviride* inoculation can increase plant stress tolerance via an increase in antioxidant production, such as lycopene. Interestingly, a high additive effect among the biostimulants was recorded. Stand-alone PE application did not statistically influence lycopene concentration, whereas, when the PE treatments were combined with other biostimulants, an increase in lycopene concentration compared to the control was recorded.

Biostimulants promoted fruit total polyphenols. As reported by Colla et al. [32] and López-Bucio et al. [58], the application of microbial and non-microbial biostimulants has a positive effect on plant secondary metabolism activity and, consequently, the biosynthesis of polyphenols. The small peptides and amino acids comprising the PE and PH had a hormone-like effect on plants, with positive outcomes on the stimulation of secondary metabolites [5]. Contrariwise, *T. atroviride* can establish a relationshup with plant roots and can produce secondary metabolites and phytohormone signaling molecules, which elicit plant secondary metabolism [36]. Finally, for total polyphenols, no synergistic effect was attributed to the biostimulants.

## 4. Materials and Methods

### 4.1. Plant Material and Experimental Site

**Figure 6:**
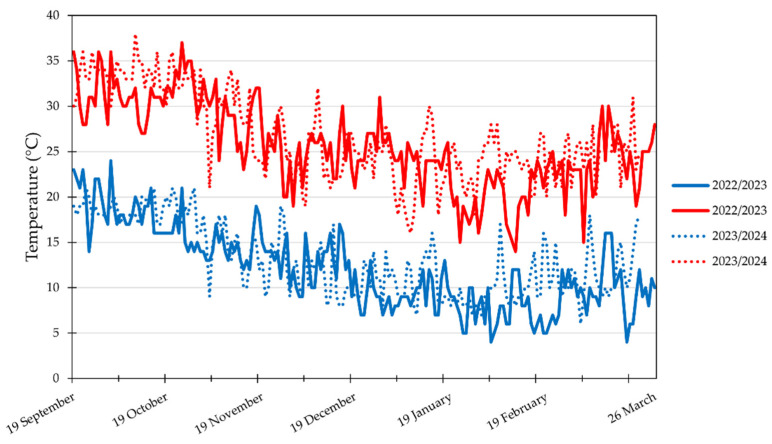
Minimum (blue color) and maximum (red color) temperatures recorded during the 2022–2023 and 2023–2024 growing cycles (from 19 September to 26 March) in the experimental greenhouse.

This study was performed in Comiso (Ragusa province, RG) on an experimental farm (36°99′64.8″ N, 14°59′88.3″ E) belonging to the lead partner of the SO.MI.PR.O.N. project, at 189 m a.s.l. The trial was carried out, for two successive cultivation cycles (2022–2023 and 2023–2024), in an unheated greenhouse (width 52 m and length 72 m) covered with a transparent polyethylene film, during the autumn–spring period. The experiment was carried out in a sandy clay soil (45.5% sand, 16.0% silt, and 38.5% clay) at pH 7.8, containing 1.8% organic matter, 0.09% total nitrogen, 6.2 mg kg^−1^ dw of P_2_O_5_, and 410.0 mg kg^−1^ dw of potassium. On 19 September (first cultivation cycle) and 25 September (second cultivation cycle), seedlings of the ‘Creativo’ F_1_ hybrid cherry tomato (*Solanum lycopersicum* L.) (HM Clause, Davis, CA, USA) were transplanted, obtaining a plant density of 3 plants m^−2^. Prior to fertilization, soil samples were collected from the experimental field and analyzed for their physico-chemical properties. All tomato plants were fertigated with 230 kg ha^−1^ of N, 150 kg ha^−1^ of P_2_O_5,_ and 250 kg ha^−1^ of K_2_O, according to the average withdrawls of tomato crops grown in Mediterranean environment conditions and the soil source. A two-branch pruning system was adopted. Throughout the growing cycles, all tomato plants were managed according to the method of Tesi [65]. Maximum and minimum temperatures inside the greenhouse were recorded via a data logger (BL30, Trotec, Heinsberg, Germany) (Figure 6).

### 4.2. Biostimulant Treatments

Three biostimulant treatments, all from the Hello Nature company (Hello Nature Italia srl, Rivoli Veronese, Italy), a *Trichoderma atroviride* (Tricho) Condor^®^, a tropical plant extract (PE) Auxym^®^, and a legume-derived protein hydrolysate (PH) Trainer^®^ were used to evaluate the single and combined effects. The day before transplantation, the tomato plants were inoculated, with the tomato root system soaked for 15 min using a dose of 6.25 g L^−1^ of Condor^®^. Root inoculation was repeated 15 and 45 days after transplantation(DAT), with 100 mL of solution supplied per plant. Every 15 days, from the beginning of the tomato flowering stage to the end of the growing cycle, the tomato plants were sprayed with PE and PH using a dosage of 1.5 mL L^−1^ and 3 mL L^−1^, respectively. For each treatment, a solution of 0.5 L m^2^ was supplied. The control plants were sprayed with only water.

### 4.3. Fruit Production and Fresh Shoot Biomass

Fruit production traits and fresh shoot biomass were recorded for all plants. Total fruit production was instantly recorded after each harvest, and it was parted into marketable and non-marketable fruit production and presented as g plant^−1^. The number of marketable fruits, presented as number plant^−1^, was recorded, and the average weight of marketable fruits, expressed as g fruit^−1^, was calculated. In comparison, fresh shoot biomass (g plant^−1^) was recorded at the end of the growing cycle.

### 4.4. Proximal Fruit Quality

At the third harvest, qualitative analyses were performed on five randomly collected fruits for each treatment. Fruit dry matter content was assessed using a ventilated oven at a temperature of 80 °C, with 500 g of the fruit sample dried until constant weight. Data were expressed as percentages.

Fruit firmness was measured using a Trsnc digital penetrometer (Forlì, Italy). On two opposite sides of the fruits’ equatorial zone, with a 3 mm stainless steel cylindrical probe, the fruits’ resistance to penetration was evaluated. The values were expressed as newtons (N). After obtaining the juice by centrifuging the tomatoes and filtering, a digital refractometer (MTD-045nD, Three-In-One Enterprises Co., Ltd., New Taipei, Taiwan) was used for the evaluation of the soluble solids content (TSS) values. The data were reported as °Brix.

Titratable acidity was calculated with Han et al.’s [66] method, using a 10 g aliquot of fruits mixed in 50 mL of distilled H_2_O and titrated to an endpoint of pH 8.1, with 0.1 N NaOH. Titratable acidity was expressed as mg L^−1^. For determination of ascorbic acid content, tomato fruits were squeezed and then measurements were performed with a reflectometer (Merck RQflex* 10, Darmstadt, Germany), using Reflectoquant Ascorbic Acid Test Strips. The value was expressed as mg of ascorbic acid per 100 g of fresh weight (mg 100 g fw^−1^).

### 4.5. Fruits’ Functional Components

Total polyphenols were estimated according to the method of Meda et al. [67], using a spectrophotometric assay, and the results were reported as mg kg^−1^ gallic acid fresh weight. Briefly, 2 g of each sample was weighed out and extracted with 50 mL of methanol. Extraction was conducted under stirring for 60 min at 60 °C. The mixture was filtered through filter paper (Whatman No. 3), filled into a 50 mL volumetric flask, and allowed to set in the dark until analysis. The standard or tomato sample extract (100 µL; in triplicate) was mixed with 0.4 mL Folin-C reagent (Sigma–Aldrich Chemie, Steinheim, Germany). After 5 min, 0.8 mL of 10% sodium carbonate (Na_2_CO_3_) was added to promote rapid oxidation of the phenolic compound, resulting in colorimetric variation. The absorbance (at 765 nm) of the solution was recorded after 2 h of incubation at room temperature and compared with a calibration curve using gallic acid (Sigma–Aldrich Chemie, Steinheim, Germany) (0–200 mg/L) as standard to produce the calibration curve. The lycopene content was established according to the protocol used by Sadler et al. [68] and expressed in mg kg^−1^ of fresh weight. Briefly, 5 g of homogenized sample was extracted by adding 50 mL of a mixture of hexane/acetone/ethanol (2:1:1, *v*/*v*/*v*) and stirred. The value was evaluated by measuring the absorbance (at 472 nm) of the lycopene hexane fraction and comparing it with a calibration curve using pure lycopene (Sigma, St. Louis, MO, USA) as standard.

### 4.6. Experimental Setup and Statistics

Eight biostimulant treatments (the control, PE, PH, Tricho, PE + PH, PE + Tricho, PH + Tricho, and PE + PH + Tricho) were employed in a randomized complete block design (RCBD), with three replications per treatment (9 plants per replicate), rendering a total of 216 plants. This experimental setup was repeated for two consecutive years (Y). All statistical analyses were performed with SPSS V. 28 software, using ANOVA and Student’s *t*-test. Separation of the mean biostimulant (B) and Y × B interaction was performed using Tukey’s HSD test (*p* ≤ 0.05) while the mean Y effect was compared using the *t*-test. Percentage data were subjected to arcsin conversion prior to ANOVA analysis (Ø = arcsin (*p*/100)^1/2^). The entire data set was also subjected to heat-map analysis through the online program package clustvis (https://biit.cs.ut.ee/clustvis/), accessed on 3 June 2024.

### 4.7. Partial Budget Analysis

A partial budget analysis was performed to evaluate the net economic benefits that may encourage cherry tomato cultivation using microbial and/or non-microbial biostimulants. The economic approach of Giordano et al. [69] and Vultaggio et al. [31] was adopted. The biostimulants’ added costs and gross returns were considered. The added net return was calculated as follows:

Added net return=Added gross return−Added variable cost

## 5. Conclusions

This study explored the application, alone or combined, of three biostimulants (tropical plant extracts, vegetal protein hydrolysate, and *Trichoderma atroviride*) on the ‘Creativo’ F1 cherry tomato hybrid. Overall, we found that the use of biostimulants increased plant performance in terms of growth, yield, and quality and boosted the nutraceutical traits of tomatoes, such as lycopene and total polyphenols. The combined application of all biostimulants (Tricho + PE + PH) was able to increase growth and yield traits, total soluble solids, ascorbic acid, lycopene, and total polyphenols, whereas it reduced fruit firmness, fruit dry matter, and titratable acidity compared to the control. In comparison, the best combinations for increasing tomato qualitative features were PE + PH, Tricho + PE, and Tricho + PH. Thus, the current study highlights that precise biostimulant combinations can promote specific traits (e.g., yield, quality, functional traits, economic profitability, etc.). The results of this study could be the basis for the definition of new biostimulant products and agronomic protocols, using microbial and non-microbial products. However, to better understand the physiological and biochemical mechanisms behind these responses, further studies including omics analyses are needed.

## Figures and Tables

**Table 1:** Analysis of variance output for all recorded parameters in response to year, biostimulant, and their interaction.

Parameters	Year (Y)	Biostimulant (B)	Y × B
	*Significance (p-value)*		
Total fruit production	n.s. (0.737)	*** (0.001)	n.s. (0.823)
Marketable fruit production	n.s. (0.768)	*** (0.001)	n.s. (0.507)
Number of marketable fruits	n.s. (0.778)	*** (0.001)	n.s. (0.787)
Average weight of marketable fruits	n.s. (0.901)	*** (0.001)	n.s. (0.632)
Fresh shoot biomass	n.s. (0.398)	*** (0.001)	*** (0.001)
Fruit dry matter	n.s. (0.592)	*** (0.001)	n.s. (0.955)
Firmness	n.s. (0.937)	*** (0.001)	n.s. (0.743)
Total soluble solids	n.s. (0.959)	*** (0.001)	n.s. (0.609)
Titratable acidity	n.s. (0.831)	*** (0.001)	*** (0.001)
Ascorbic acid	n.s. (0.597)	*** (0.001)	* (0.013)
Total polyphenols	n.s. (0.937)	*** (0.001)	n.s. (0.740)
Lycopene	n.s. (0.774)	*** (0.001)	** (0.004)

Biostimulant (B) effect and Y × B interaction were determined using Tukey’s HSD test (*p* ≤ 0.05) while the mean Y effect was compared using the *t*-test. n.s.: not significant; *: significant at *p* ≤ 0.05; **: significant at *p* ≤ 0.01; ***: significant at *p* ≤ 0.001.

**Table 2:** Total fruit production, marketable fruit production, number of marketable fruits, and average weight of marketable fruit as influenced by cultivation year, biostimulant, and their interaction.

P.T.	Total Fruit Production	Marketable Fruit Production	Number of Marketable Fruits	Average Weight of Marketable Fruit
	[g pt^–1^]		[n° pt^–1^]	[g]
*Year (Y)*				
I	1648.7	1513.8	109.6	13.9
II	1667.2	1531.0	110.9	14.0
*Biostimulant (B)*				
Control	1379.1 f	1163.0 d	105.2 b	11.1 c
PE	1904.2 a	1727.0 a	141.5 a	12.2 bc
PH	1490.5 e	1369.4 c	108.5 b	12.8 bc
Tricho	1581.5 cd	1475.7 b	106.2 b	14.0 b
PE + PH	1800.9 b	1686.3 a	98.1 b	17.3 a
Tricho + PE	1548.6 de	1454.3 bc	108.1 b	13.5 bc
Tricho + PH	1656.6 c	1524.9 b	108.9 b	14.1 b
Tricho + PE + PH	1902.5 a	1778.5 a	105.7 b	17.0 a
*Significance*				
Y	n.s	n.s	n.s	n.s
B	***	***	***	***
Y × B	n.s	n.s	n.s	n.s

Data are presented as the mean ± SD. Separation of the mean biostimulant (B) effect was performed using Tukey’s HSD test (*p* ≤ 0.05) while the mean year (Y) effect was compared using the *t*-test. ***: significant at *p* ≤ 0.001; n.s: not statistically significant. Different letters indicate significantly different means. First year: 2022–2023; second year: 2023–2024; Control: non-treated plants; PE: tropical plant extract; PH: legume-derived protein hydrolysate; Tricho: *Trichoderma atroviride*.

**Table 3:** Firmness, TSS, and fruit dry matter as influenced by cultivation year, biostimulant, and their interaction.

P.T.	Firmness	TSS	Fruits Dry Matter
	N	°Brix	%
*Year (Y)*			
I	26.9	8.7	9.0
II	26.8	8.7	9.1
*Biostimulant (B)*			
Control	22.5 d	7.0 d	9.7 a
PE	25.3 c	8.7 b	8.7 de
PH	27.3 bc	9.7 a	9.3 abc
Tricho	29.9 a	9.7 a	9.4 ab
PE + PH	28.9 ab	8.1 c	8.9 cde
Tricho + PE	29.6 ab	8.7 b	8.8 de
Tricho + PH	28.7 ab	8.9 b	9.6 a
Tricho + PE + PH	22.7 d	8.8 b	8.5 e
*Significance*			
Y	n.s	n.s	n.s
B	***	***	***
Y × B	n.s	n.s	n.s

Data are presented as the mean ± SD. Separation of the mean biostimulant (B) effect was performed using Tukey’s HSD test (*p* ≤ 0.05) while the mean year (Y) effect was compared using the *t*-test.; n.s: not statistically significant; ***: significant at *p* ≤ 0.001. Different letters indicate significantly different means. First year: 2022–2023; second year: 2023–2024; Control: non-treated plants; PE: tropical plant extract; PH: legume-derived protein hydrolysate; Tricho: *Trichoderma atroviride*.

**Table 4:** Added returns of cherry tomato grown under greenhouse conditions achieved by tropical plant extract (PE), plant protein hydrolysate (PH), *Trichoderma atroviride* (Tricho), or their combinations compared to the untreated control.

Biostimulant	Yield Increase (kg ha^−1^)	Price (EUR kg^−1^)	Added Gross Return (EUR ha^−1^)	Added Variable Cost (EUR ha^−1^)			Total (EUR ha^−1^)	Added Net Return (EUR ha^−1^)
				Biostimulant Treatment	Application	Harvest		
PE	18,612.0	2.0	37,224.0	4875.0	100	4094.6	9069.6	28,154.4
PH	6811.2	2.0	13,622.4	1800.0	100	1498.5	3398.5	10,223.9
Tricho	10,319.1	2.0	20,638.2	9270.0	100	2270.2	11,640.2	8998.0
PE + PH	17,268.9	2.0	34,537.8	6675.0	100	3799.2	10,574.2	23,963.6
Tricho + PE	9612.9	2.0	19,225.8	14,145.0	200	2114.8	16,459.8	2766.0
Tricho + PH	11,942.7	2.0	23,885.4	11,070.0	200	2627.4	13,897.4	9988.0
Tricho + PE + PH	20,311.5	2.0	40,623.0	15,945.0	200	4468.5	20,613.5	20,009.5

The costs of biostimulants were defined by the suppliers (PE = 65 EUR L^−1^; PH = 12 EUR L^−1^; Tricho = 150 EUR kg^−1^); the costs of biostimulant applications were considered reputable based on the information provided by local agricultural contractors.

## Data Availability

The data produced in this research are available upon request due to privacy restrictions.
